# Pre- and Postnatal Fine Particulate Matter Exposure and Renal Fibrogenesis in Adult Male Rats: The Role of Vitamin D Supplementation

**DOI:** 10.3390/cimb47060387

**Published:** 2025-05-22

**Authors:** Min-Hwa Son, Hyung-Eun Yim, Yu-Seon Lee, Yoon-Jeong Nam, Ju-Han Lee

**Affiliations:** 1 Department of Pediatrics, Korea University Ansan Hospital, 123, Jeokgeum-ro, Danwon-gu, Ansan-si 15355, Gyeonggi-do, Republic of Korea; lsminhwa@gmail.com; 2 Medical Science Research Center, Korea University Ansan Hospital, 123, Jeokgeum-ro, Danwon-gu, Ansan-si 15355, Gyeonggi-do, Republic of Korea; useon0926@naver.com (Y.-S.L.); nyj90504@naver.com (Y.-J.N.); 3 Department of Pathology, Korea University Ansan Hospital, 123, Jeokgeum-ro, Danwon-gu, Ansan-si 15355, Gyeonggi-do, Republic of Korea; repath@korea.ac.kr

**Keywords:** chronic kidney disease, environmental pollution, fetal kidney development, fine dust

## Abstract

Prolonged exposure to fine particulate matter (PM_2.5_) has been implicated in accelerated aging, including organ fibrosis. This study aimed to investigate whether prenatal and postnatal PM_2.5_ exposure promotes renal fibrogenesis in adulthood and whether long-term vitamin D supplementation alleviates associated renal injury. Pregnant Sprague-Dawley rats were randomly assigned to three groups: control (normal saline, NS), PM_2.5_ exposure, and PM_2.5_ exposure with vitamin D supplementation during gestation and lactation (*n* = 3/group). Male offspring were subsequently exposed to the same conditions from postnatal weeks 3 to 8 (*n* = 7/group). On postnatal day 56, PM_2.5_-exposed rats showed lower body weight and more severe glomerular and tubulointerstitial damage compared to controls. Serum calcium levels were elevated in the PM_2.5_ group. The expression of intrarenal renin, transforming growth factor-β1, α-smooth muscle actin, and vimentin was upregulated, accompanied by increased collagen deposition. Long-term vitamin D supplementation reversed most of these changes, except for intrarenal vimentin expression and serum calcium levels. These findings indicate that prenatal and postnatal PM_2.5_ exposure can activate intrarenal renin signaling and fibrogenic pathways, contributing to renal fibrosis later in life. Long-term vitamin D supplementation may provide partial protective effects against PM_2.5_-induced renal fibrogenesis.

## 1. Introduction

Particulate matter (PM) with an aerodynamic diameter of less than 2.5 μm (PM_2.5_) is a major contributor to the global burden of disease [1,2]. Emerging evidence has identified an association between long-term PM_2.5_ exposure and an increased risk of chronic kidney disease (CKD) [3]. PM_2.5_ is known to negatively impact on the kidney through diverse mechanisms such as inflammation, oxidative stress, DNA damage, disruption of the renin–angiotensin system (RAS), and endothelial dysfunction [4]. Furthermore, PM_2.5_ has been reported to promote fibrosis by initiating abnormal epithelial–mesenchymal transition (EMT) [5].

EMT is a biological process in which epithelial cells lose their epithelial characteristics and acquire mesenchymal properties [6,7]. It is characterized by a loss of adhesion between epithelial cells, the expression of mesenchymal cell markers such as α-smooth muscle actin (α-SMA) and vimentin, and reorganization of the intracellular actin cytoskeleton [8]. During EMT, the epithelial cell basement membrane, which separates epithelial cells from the interstitium, is degraded. This degradation facilitates the migration and infiltration of mesenchymal cells into the interstitial tissue. Several growth factors and cytokines are involved in EMT, with transforming growth factor-β1 (TGF-β1) being the main inducer [6,8]. Angiotensin II, a key component of the RAS, is also implicated in inducing EMT through both TGF-β1-dependent and -independent mechanisms [9]. EMT can occur in a partial state, characterized by co-expression of epithelial and mesenchymal markers, reflecting its dynamic and context-dependent nature in renal pathology [10].

The homeostasis of the RAS is crucial for both fetal renal development and the progression of CKD [11,12]. Maternal vitamin D deficiency during pregnancy disrupts fetal RAS homeostasis, leading to persistent imbalances that increase the risk of CKD and hypertension later in life [13,14]. In our previous in vitro and in vivo studies [15,16], we demonstrated that PM_2.5_ exposure induced renal injury, accompanied by disturbances in vitamin D signaling and the RAS pathway. Specifically, maternal PM_2.5_ exposure led to increased glomerular and tubular injury, oxidative stress, and inflammation in the kidneys of both rat dams and their offspring. These effects were alleviated by maternal vitamin D supplementation [16]. In this study, we hypothesized that chronic PM_2.5_ exposure promotes renal fibrogenesis later in life and that long-term vitamin D supplementation attenuates renal injury in adult offspring rats. We aimed to explore whether pre- and postnatal PM_2.5_ exposure induces renal fibrosis through abnormal EMT in adulthood. We further evaluated the protective effects of vitamin D supplementation on the molecular pathways underlying renal damage caused by prolonged PM_2.5_ exposure.

## 2. Materials and Methods

### 2.1. PM Collection, Analysis, and Preparation

Airborne PM samples were collected as described in the previous experiment [15,16]. Using a large-capacity air sampler (HV-1700RW, Sibata, Tokyo, Japan) with a flow rate of 1000 L/min, we collected fine particles on the rooftop of Korea University Ansan Hospital, located in Ansan-si, Gyeonggi-do, Republic of Korea. Five quartz fiber filters (QR-100, Sibata, Tokyo, Japan) were used to capture PM over 2 days (24 to 25 December 2019). Real-time air quality data (https://www.airkorea.or.kr/eng (accessed on 25 April 2022)) were referenced to determine the concentration of PM_2.5_. To obtain a PM suspension, the filter attached to the atmospheric particles was dried in an automatic dryer (Sanpla Dry Keeper, Sanplec Corp., Osaka, Japan) before PM was extracted. Subsequently, the filters were cut into small pieces each measuring about 2 × 2 cm^2^ and immersed in 100 mL of phosphate buffered saline, followed by sonication three times for 30 min. After separating the PM suspension on a quartz fiber filter by shaking for 10 min, the PM suspension was filtered through a 0.2 μm syringe filter to remove larger interfering particles. Ionic PM_2.5_ components were analyzed by ion chromatography (Eco, Metrohm AG, Herisau, Switzerland) and water-soluble organic carbon (WSOC) was quantified using a total organic carbon analyzer (TOC-L, Shimadzu Co., Kyoto, Japan) at the Korea Basic Science Institute Seoul Center.

As in the previous study, the following equation, adapted from the average daily dose calculation of the United States Environmental Protection Agency, was used to determine the exposure to PM_2.5_ in preparation for the animal study [17]:

Average daily dose (mg/kg·day) = (Cair × InhR × ET × EF × ED)/(BW × AT).

where Cair, InhR, ET, EF, ED, BW, and AT represent the concentration of the contaminant in the air (mg/m^3^), inhalation rate (m^3^/day), exposure time (h/day), exposure frequency (day/year), exposure duration (year), body weight (kg), and average time (day), respectively. To induce maternal exposure to PM_2.5_, dams were administered 107 μL of PM_2.5_ suspension once daily for a month (a total of 21 doses), resulting in a total dose of 2.247 mL (107 μL × 21 doses = 2.247 mL). An average person (weighing 60 kg) has a tidal volume of 500 mL, a respiratory rate of 14 breaths per minute, and a total respiratory volume of 9660 L/day over 23 h [17]. The air sampler used in the study had a suction flow of 1,380,000 L/day (1000 L/min × 23 h), with airborne particles collected for 23 h and a 1 h rest period. Based on this, the airflow extracted by the air sampler for five filters corresponded to 714 days of human respiration (1,380,000 L/day × 5 filters ÷ 9660 L/day = 714 days). The particles collected on these five filters over two days were extracted using 200 mL of normal saline (NS). Consequently, 0.28 mL of PM_2.5_ suspension represented the estimated daily PM_2.5_ exposure for a person (200 mL ÷ 714 days = 0.28 mL/day). The total dose administered to each dam (2.247 mL) was therefore equivalent to eight days of human PM_2.5_ exposure (2.247 mL ÷ 0.28 mL/day = 8 days). Meanwhile, offspring rats were exclusively breastfed by their mothers, which were administered either NS, PM_2.5_, or PM_2.5_ with vitamin D supplementation until 3 weeks postpartum. From 3 to 8 weeks of age, offspring rats received the same treatment as their mothers (NS, PM_2.5_, or PM_2.5_ with vitamin D) for a total of 14 doses. Each offspring rat was administered 180 μL of PM_2.5_ per day, amounting to a total dose of 2.52 mL (180 μL × 14 doses = 2.52 mL). Given that 0.28 mL of PM_2.5_ suspension represents one day of human exposure, the total dose administered to each offspring rat corresponded to approximately 9 days of human PM_2.5_ exposure (2.52 mL ÷ 0.28 mL/day = 9 days).

### 2.2. Animal Preparation

**Figure 1:**
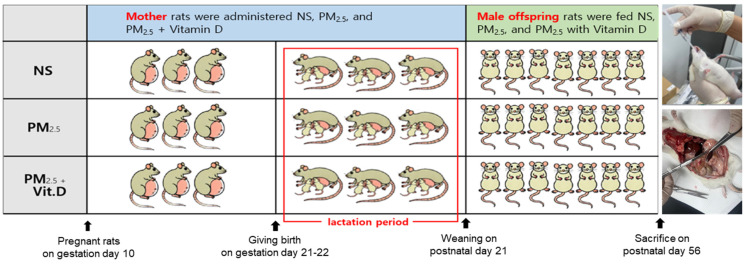
Schematic representation of the experimental procedure. NS, normal saline; PM_2.5_, fine particulate matter.

Nine pregnant Sprague-Dawley rats (Raonbio Co., Ltd., Yongin-si, Republic of Korea) on gestation day 10 were reared in standard housing conditions. Pelleted food (Purina, Sung-nam, Republic of Korea) for experimental rodents and filtered tap water were provided ad libitum. Pregnant rats were divided into three groups: control, PM_2.5_, and PM_2.5_ with vitamin D (*n* = 3/group). Dams were allowed to deliver their offspring naturally on gestation days 21–22. They were fed with NS (107 μL, 5 times/week), PM_2.5_ (107 μL dissolved in saline, 5 times/week), and PM_2.5_ (107 μL dissolved in saline, 5 times/week) with vitamin D (cholecalciferol, 1000 IU/kg, 3 times/week; FND Net Co., Seoul, Republic of Korea) via an orogastric tube from gestation day 11 to lactation day 21 (the period of nephrogenesis in offspring rats) according to the assigned group. The offspring rats were exclusively breastfed by their dams, who were fed NS, PM_2.5_, or PM_2.5_ with vitamin D with no other intervention until postnatal day 21. On postnatal day 22, seven male offspring rats were selected among the offspring born to dams treated with NS, PM_2.5_, or PM_2.5_ with vitamin D. From 3 to 8 weeks of age, the selected male offspring rats were fed NS (180 μL, 3 times/week), PM_2.5_ (180 μL dissolved in saline, 3 times/week), and PM_2.5_ (180 μL dissolved in saline, 3 times/week) with vitamin D (cholecalciferol, 1000 IU/kg, 3 times/week; FND Net Co.). Only male offspring were used because studies with animal models have shown that they are more susceptible to fetal injury during development [18,19]. The offspring rats were then anesthetized with 2% isoflurane, and their blood was collected via cardiac puncture. The kidneys were then harvested and processed (Figure 1).

### 2.3. Biochemical Analysis

At 8 weeks of age, serum levels of 25(OH)D, Ca^2+^, and cystatin C were determined using commercial enzyme-linked immunosorbent assay (ELISA) kits. Specifically, 25(OH)D ELISA kit (MyBioSource, San Diego, CA, USA), calcium assay kit (Abcam, Waltham, MA, USA), and rat cystatin C quantikine ELISA kit (R&D systems, Minneapolis, MN, USA) were utilized, respectively.

### 2.4. Histological Examination

Rat kidneys were formalin-fixed, dehydrated, embedded into the paraffin, and cut into slices 4 μm in thickness. Hematoxylin and eosin staining and Masson’s trichrome (MT) staining were performed. Glomerular damage and tubulointerstitial injury were scored as in the previous studies [16,20]. Tubulointerstitial fibrosis was also quantified as the ratio of the positively stained area (blue) to the total area: 0, 0%; 1, <25%; 2, 25–50%; 3, 50–75%; and 4, >75%. Ten random fields per kidney section (×200 magnification) were used for quantification in a blinded manner. All stained slides were digitized using the Pannoramic Scan II system (3DHISTECH; Sysmex, Budapest, Hungary) and analyzed on digital images using CaseViewer software (3DHISTECH, version 2.2.0).

### 2.5. Western Blotting

Protein extractions and Western blotting were conducted following previously described methods [16]. Membranes were incubated with primary antibodies overnight at 4 °C: Vitamin D receptor (VDR) (Abcam), Klotho (Invitrogen, Carlsbad, CA, USA), cytochrome P450 mixed-function oxidase (CYP) 27B1(Abcam), and CYP24A1 (Invitrogen), renin (Santa Cruz Biotechnology, Santa Cruz, CA, USA), angiotensin-converting enzyme (ACE)(Invitrogen), angiotensin II type 1 receptor (AT1R) (Invitrogen), α-SMA (Abcam), vimentin (Santa Cruz Biotechnology), TGF-β1 (Santa Cruz Biotechnology), and E-cadherin (Santa Cruz Biotechnology). The blots were then incubated with horse radish peroxidase-conjugated anti-rabbit or anti-mouse immunoglobulin G (Cell Signaling Technology, Danvers, MA, USA) at room temperature for 2 h. The ChemiDoc Touch Imaging System (Bio-Rad Laboratories, Hercules, CA, USA) was used for imaging of Western blots. The intensity of the identified lanes was quantified using a densitometer. The level of each protein was expressed relative to that of β-actin (Santa Cruz Biotechnology) or GAPDH (Santa Cruz Biotechnology).

### 2.6. Immunohistochemistry

The paraffin-embedded kidney sections were deparaffinized, rehydrated, and antigen-retrieved. Antigen retrieval was performed using tris-ethylenediaminetetraacetic acid buffer solution with a pH 8.0, and all slides were stained with Dako AutoStainer (Dako, Santa Clara, CA, USA). Primary antibodies against renin (Santa Cruz Biotechnology), α-SMA (Cell Signaling Technology), vimentin (Cell Signaling Technology), and TGF-β1 (Novus, Centennial, CO, USA) were used. To visualize primary antibodies, the Novolink Polymer Detection System (RE7150-K; Leica Biosystems, Newcastle Upon Tyne, UK) was utilized according to the manufacturer’s instructions. The slides were then visualized through Pannoramic Scan II (3DHISTECH; Sysmex).

### 2.7. Statistical Analysis

All experiments were performed at least three times. The results are presented as means ± standard error of the mean (SEM). Statistical analysis was performed using GraphPad Prism ver. 7.0. All data were analyzed using one-way analysis of variance followed by Tukey’s post hoc test. *p*-values of <0.05 were considered statistically significant.

## 3. Results

### 3.1. Characteristics of PM_2.5_

The PM_2.5_ samples used in this study were collected over 2 days with PM_2.5_ concentrations of 51 μg/m^3^ and 36 μg/m^3^. Table 1 represents the concentration of PM_2.5_ in the air by converting the liquid ppm concentration, which was analyzed by extracting PM_2.5_ collected from 200 mL of saline solution, into μg/m^3^. The concentration of NO_3_^−^ (nitrate) was the highest, followed by that of WSOC, NH_4_^+^ (ammonium), SO_4_^2−^ (sulfate), etc.

### 3.2. Body Weight Changes and Biochemical Data

**Figure 2:**
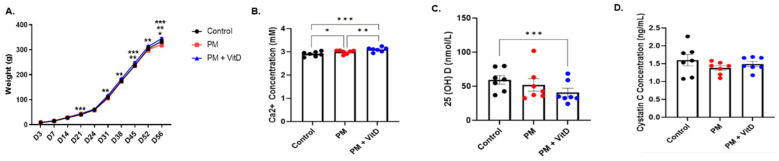
Body weight changes and biochemical data. (**A**) Weights from postnatal days 3 to 56. (**B**) Serum Ca^2+^ concentration. (**C**) Serum 25(OH)D level. (**D**) Serum cystatin C concentration (*n* = 7 for each group) (* *p* < 0.05, Control vs. PM_2.5_; ** *p* < 0.05, PM_2.5_ vs. PM_2.5_+VitD; *** *p* < 0.05, Control vs. PM_2.5_+VitD). PM, particulate matter; VitD, vitamin D. Data are presented as mean ± SEM. Statistical analysis was performed using one-way analysis of variance followed by Tukey’s post hoc test.

The body weights of male offspring rats in the control, PM_2.5_, and PM_2.5_ with vitamin D groups were measured from postnatal days 3 to 56. At birth, there were no differences in body weights among the groups. On postnatal day 21, the body weights of rats in the PM_2.5_ with vitamin D group were higher than those in the control group. On postnatal day 56, the PM_2.5_ group showed the lowest body weights, which were significantly smaller than those of the control group. From postnatal days 31 to 56, the body weights of rats in the PM_2.5_ with vitamin D group were consistently higher than those in the PM_2.5_ group (Figure 2A). Serum Ca^2+^ levels were elevated in the PM_2.5_ and PM_2.5_ with vitamin D groups compared to the control group. Among the groups, the PM_2.5_ with vitamin D group had the highest levels of serum Ca^2+^ (Figure 2B). In contrast, the levels of serum 25(OH)D showed an opposite pattern relative to Ca^2+^ levels. The concentrations of 25(OH)D were lower in the PM_2.5_ with vitamin D group than in the control group (Figure 2C). No significant differences were observed in serum cystatin C concentrations among the three groups (Figure 2D).

### 3.3. Renal Histological Alterations

**Figure 3:**
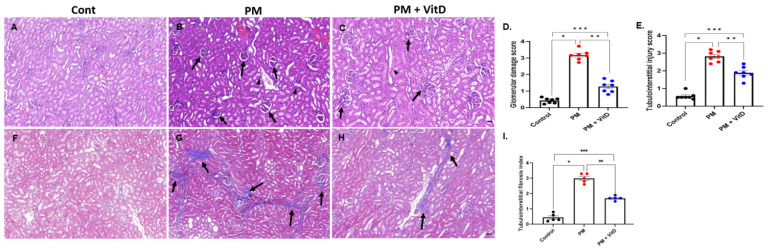
Renal histological alterations in adult offspring rats. (**A**–**C**) Hematoxylin and eosin stain (arrows, sclerotic glomeruli; arrowheads, degenerated or dilated tubules) (×100, bar = 100 µm). (**D**) Semi-quantitative analysis of glomerular damage score. (**E**) Semi-quantitative analysis of tubulointerstitial injury score. (**F**–**H**) Masson’s trichrome stain (arrows, interstitial collagen) (×100, bar = 100 µm). (**I**) Semi-quantitative analysis of tubulointerstitial fibrosis index (*n* = 5–7 for each group) (* *p* < 0.05, Control vs. PM_2.5_; ** *p* < 0.05, PM_2.5_ vs. PM_2.5_+VitD; *** *p* < 0.05, Control vs. PM_2.5_+VitD). Cont, control; PM, particulate matter; VitD, vitamin D. Data are presented as mean ± SEM. Statistical analysis was performed using one-way analysis of variance followed by Tukey’s post hoc test.

Glomerular damage, which includes mesangial expansion and destructive glomerular tufts, and tubulointerstitial injury, such as tubular dilatation and cast formation, were more frequent in the PM_2.5_ group compared to the control group. The severity scores for glomerular damage and tubulointerstitial injury were significantly higher in the PM_2.5_ group than in the control group. Vitamin D treatment reduced the extent of these injuries (Figure 3A–E). Moreover, MT staining revealed a greater deposition of blue-stained collagen fibers in the PM_2.5_ group, suggesting increased renal tubulointerstitial fibrosis due to prolonged PM_2.5_ exposure. Vitamin D treatment lessened the extent of these fibrotic changes (Figure 3F–I).

### 3.4. Renin, ACE, and AT1R Expression

**Figure 4:**
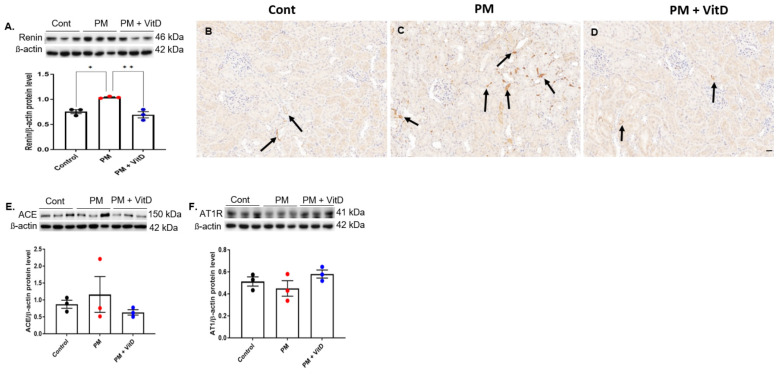
Intrarenal RAS signaling in adult offspring rats. (**A**) Renin. (**B**–**D**) Immunohistochemistry for renin expression (arrows) (×200, bar = 50 µm). (**E**) ACE. (**F**) AT1R (*n* = 3 for each group) (* *p* < 0.05, Control vs. PM_2.5_; ** *p* < 0.05, PM_2.5_ vs. PM_2.5_ +VitD). RAS, renin angiotensin system; ACE, angiotensin converting enzyme; AT1R, angiotensin II type 1 receptor; Cont, control; PM, particulate matter; VitD, vitamin D. Immunohistochemistry was performed using a mouse monoclonal anti-renin antibody (Santa Cruz, sc-133145; 1:200). Antibody specificity was validated by known positive control tissues and by omitting the primary antibody as a negative control. Data are presented as mean ± SEM. Statistical analysis was performed using one-way analysis of variance followed by Tukey’s post hoc test.

Since intrarenal RAS activation contributes to both renal pathophysiological processes and kidney development, we assessed changes in RAS components in the kidneys of adult offspring. Intrarenal renin expression increased in the PM_2.5_ group, and this increase was reversed by vitamin D treatment (Figure 4A). Immunohistochemical analysis revealed strong renin localization, predominantly in the renal interstitium, in the PM_2.5_ group. Vitamin D treatment reduced intrarenal renin activity (Figure 4B–D). The protein expression levels of ACE and AT1R did not show differences among the three groups (Figure 4E,F).

### 3.5. VDR, Klotho, CYP27B1, and CYP24A1 Expression

**Figure 5:**
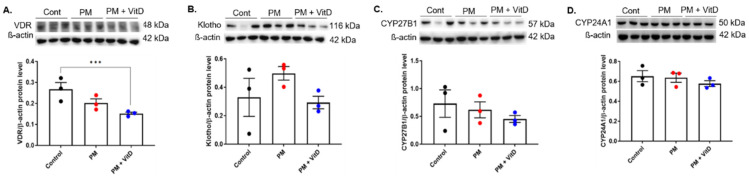
Intrarenal vitamin D signaling in adult offspring rats. (**A**) VDR. (**B**) Klotho. (**C**) CYP27B1. (**D**) CYP24A1 (*n* = 3 for each group) (*** *p* < 0.05, Control vs. PM_2.5_+VitD). VDR, vitamin D receptor; CYP, cytochrome P450 mixed-function oxidase; Cont, control; PM, particulate matter; VitD, vitamin D. Data are presented as mean ± SEM. Statistical analysis was performed using one-way analysis of variance followed by Tukey’s post hoc test.

To investigate changes in vitamin D signaling during exposure to PM_2.5_ with or without vitamin D, the expression levels of VDR, Klotho, CYP27B1, and CYP24A1 were analyzed using Western blotting. The results showed a decreasing trend of VDR expression following exposure to PM_2.5_, and exposure to PM_2.5_ with vitamin D further reduced the expression of VDR, with a statistical difference in levels between the control and PM_2.5_ with vitamin D groups (*p* < 0.05) (Figure 5A). In contrast, the protein expressions of Klotho, CYP27B1, and CYP24A1 were not different among the groups (Figure 5B–D).

### 3.6. EMT Markers and TGF-β1 Expression

**Figure 6:**
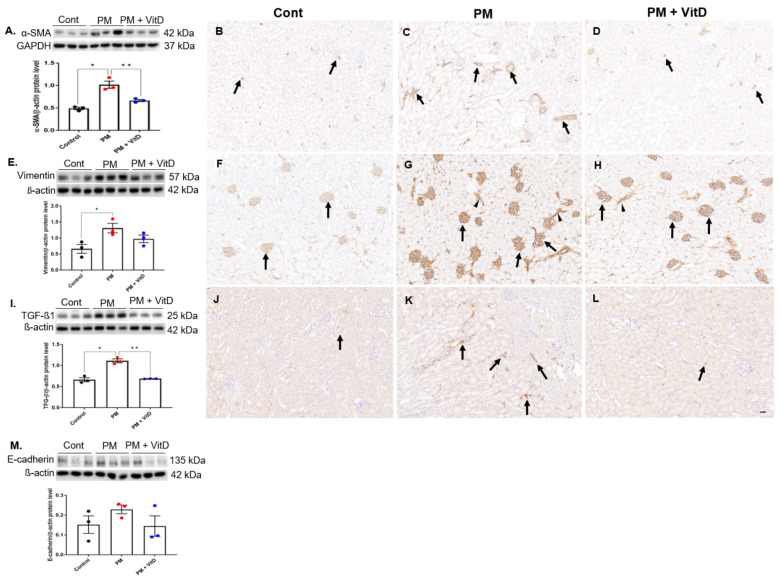
Intrarenal EMT markers. (**A**) α-SMA. (**B**–**D**) Immunohistochemistry for α-SMA expression (arrows). (**E**) Vimentin. (**F**–**H**) Immunohistochemistry for vimentin expression (arrows, glomerular capillary loops; arrowheads, peritubular vessels and interstitium). (**I**) TGF-ß1. (**J**–**L**) Immunohistochemistry for TGF-ß1 expression (arrows). (**M**) E-cadherin (*n* = 3 for each group) (×100, bar = 100 µm) (* *p* < 0.05, Control vs. PM_2.5_; ** *p* < 0.05, PM_2.5_ vs. PM_2.5_ +VitD). EMT, epithelial–mesenchymal transition; α-SMA, α-smooth muscle actin; TGF-β1, transforming growth factor-β1; Cont, control; PM, particulate matter; VitD, vitamin D. Immunohistochemistry was performed using a mouse monoclonal anti-α-SMA antibody (Cell Signaling, 19245S; 1:400), a mouse monoclonal anti-vimentin antibody (Cell Signaling, 19245S; 1:800), and a mouse monoclonal anti-TGF-β1 antibody (Novus, NBP2-22114; 1:300). Antibody specificity was validated by known positive control tissues and by omitting the primary antibody as a negative control. Data are presented as mean ± SEM. Statistical analysis was performed using one-way analysis of variance followed by Tukey’s post hoc test.

Next, we investigated the activities of EMT markers and their inducer, TGF-β1, in the kidneys. Intrarenal protein levels of α-SMA, vimentin, and TGF-β1 increased in the PM_2.5_ group (Figure 6A–L). Vitamin D administration reversed the increases in α-SMA and TGF-β1 levels (Figure 6A,I). Although vimentin levels showed a decreasing trend in the PM_2.5_ with vitamin D group, the difference was not statistically significant (Figure 6E). Immunostaining indicated a clear increase in α-SMA, vimentin, and TGF-β1 expression in the PM_2.5_ group. Specifically, α-SMA was localized in the interstitium and peritubular cells, vimentin in glomerular and interstitial cells, and TGF-β1 in the interstitium. In the PM_2.5_ with vitamin D group, the intrarenal expressions of α-SMA and TGF-β1 decreased compared to the PM_2.5_ group. In contrast, the vimentin expression in the PM_2.5_ with vitamin D group was similar to that observed in the PM_2.5_ group. E-cadherin activity remained unchanged across all groups (Figure 6M).

## 4. Discussion

This experimental study aimed to determine whether kidney damage resulting from PM_2.5_ exposure during the prenatal and postnatal period has consequences in adulthood. At 8 weeks after birth, glomerular damage and tubulointerstitial fibrogenic changes increased in the PM_2.5_ group, and vitamin D administration ameliorated the extent of this injury. Intrarenal renin expression increased in the PM_2.5_ group, and vitamin D supplementation offset this increase. Levels of α-SMA, vimentin, and TGF-β1 in kidneys increased with exposure to PM_2.5_, and vitamin D administration reduced the increases in α-SMA and TGF-β1 levels. These findings suggest that prenatal and postnatal long-term exposure to airborne PM_2.5_ can activate intrarenal renin and fibrogenic processes later in life. Long-term vitamin D supplementation may lessen PM_2.5_-induced fibrogenic renal changes in adulthood.

Human kidney development is completed in utero by gestational week 36, whereas nephrogenesis in rodents begins at embryonic day 12 and ends approximately 20 days after birth [21]. Since eight weeks of age in rats is roughly equivalent to the beginning of adulthood in humans [22], the period of PM_2.5_ exposure with or without vitamin D in our experiment corresponds to the time between the start of nephrogenesis and that of adulthood. In the present study, there were no significant differences in body weights among the groups at birth, whereas the PM_2.5_ group exhibited the lowest body weight on postnatal day 56. The PM_2.5_ group supplemented with vitamin D displayed the highest weight among the three groups. From postnatal days 31 to 56, the PM_2.5_ group supplemented with vitamin D showed consistently higher body weights compared to the PM_2.5_ group. These findings contrast with epidemiologic studies suggesting that PM_2.5_ exposure increases the risk of overweight and obesity [23]. This discrepancy may arise from differences in study design, as most human studies are short-term and cross-sectional, while this animal study examined the effects of prolonged PM_2.5_ exposure from the prenatal stage to adulthood. Considering that prenatal PM_2.5_ exposure has been associated with increased rates of preterm birth and low birth weight [24,25], the observed effects on weight may vary based on the duration of PM_2.5_ exposure and the period of evaluation. Nonetheless, PM_2.5_ exposure appears to be associated with metabolic alterations as vitamin D supplementation restored body weight in offspring rats exposed to PM_2.5_. This finding is consistent with our previous in vitro study [15], where vitamin D supplementation improved cell viability suppressed by PM_2.5_ exposure.

While the previous experiment [16] showed that exposure to PM_2.5_ during the nephrogenic period decreased renin and ACE in the offspring rats, continued exposure to PM_2.5_ into adulthood resulted in a significant increase in renin and a rising trend in ACE levels. Our earlier in vitro study [15] also revealed that PM_2.5_ exposure upregulated renin, ACE, and AT1R expression in human proximal renal tubular cells while reducing VDR expression. In line with these findings, Zhang et al. [26] reported short-term PM_2.5_ exposure in mice induced acute kidney damage, excessive oxidative stress, and inflammatory responses, along with the overexpression of ACE and AT1R activity in kidney tissue. They speculated that PM_2.5_-induced activation of the RAS could contribute to oxidative stress and inflammation. In contrast, the present study found no significant changes in ACE or AT1R levels. This discrepancy may be attributed to compensatory mechanisms involving the bradykinin system, systemic RAS, other opposing RAS components, or an insufficient dose–response relationship in individual rats. Prolonged PM_2.5_ exposure may induce distinct molecular events at various stages of the pathway. Nevertheless, given that RAS plays a key role in the pathophysiology of kidney diseases [11,27], renin activation likely contributes to kidney injury following chronic PM_2.5_ exposure. Notably, the pro-renin receptor (PRR) has been identified as a positive regulator in renal fibrosis [28,29]. PRR and its soluble form function not only as important activators of the local RAS but also activate angiotensin II-independent signaling pathways, influencing various pathophysiological processes, including cardiovascular and metabolic disorders [30]. Soluble PRR was both necessary and sufficient for TGF-β-induced fibrotic responses in cultured renal tubular cells [28]. In support of this hypothesis, PM_2.5_ exposure in this study exacerbated glomerular and tubulointerstitial damage with pronounced fibrotic changes. Prolonged PM_2.5_ exposure also increased the expression of fibrogenic molecules, which were lessened by vitamin D administration, alongside a reduction in intrarenal renin activity.

In addition, long-term PM_2.5_ exposure was associated with elevated serum Ca^2+^ levels in adult male rats. Since PM_2.5_ contains Ca^2+^ as a major cation, cellular absorption or entry into the bloodstream may activate pro-EMT Ca^2+^-dependent signaling pathways [5,31]. Supporting this hypothesis, chronic low-dose PM_2.5_ exposure has been shown to induce an EMT-like phenotype in pulmonary epithelial cells, with a subset of differentially expressed genes linked to cellular response to calcium ions [32]. Moreover, calcium-binding proteins responding to elevated Ca^2+^ have been implicated in aggravating renal fibrosis associated with EMT [33]. These findings suggest that calcium may serve as a molecular trigger of PM_2.5_-induced EMT, contributing to fibrotic kidney injury [5]. Contrary to expectations, vitamin D supplementation did not reverse serum Ca^2+^ or 25(OH)D levels, nor restore intrarenal VDR activity, implying that sustained hypercalcemia may override the regulatory effects of vitamin D signaling. In our previous study [16], maternal PM_2.5_ exposure reduced intrarenal VDR expression and serum calcium levels in offspring rats at weaning. Prolonged PM_2.5_ exposure in the present study further impaired the vitamin D signaling pathway, as evidenced by the persistent downregulation of renal VDR expression and reduced serum 25(OH)D levels, despite continued vitamin D supplementation. This indicates a disruption of the vitamin D–VDR–calcium axis, potentially driven by pollutant-induced calcium influx or endocrine disruption. Elevated calcium levels may also exert negative feedback on VDR expression and vitamin D metabolism. These in vivo findings contrast with our previous in vitro data showing VDR restoration by vitamin D [15], highlighting the complexity of systemic regulation in vivo. Collectively, these results suggest that PM_2.5_-induced hypercalcemia impairs vitamin D signaling and may limit the therapeutic benefit of vitamin D supplementation under chronic PM_2.5_ exposure.

EMT is an important process in physiological tissue repair. However, when uncontrolled and persistent, EMT can lead to fibrosis [7]. TGF-β1 serves as the primary inducer of EMT by suppressing the epithelial marker E-cadherin and promoting mesenchymal markers such as α-SMA and vimentin [7,33]. These molecular changes play a significant role in the progression of renal fibrosis, emphasizing the central role of EMT in pathological tissue remodeling. Moreover, the established crosstalk between EMT, oxidative stress, and apoptotic signaling pathways—including mitochondrial caspase activation and Bcl-2 family regulation—suggests that apoptotic mechanisms may contribute to PM_2.5_-induced renal fibrogenesis [34]. Interestingly, the EMT process may not always be complete [10]. In some cases, cells exhibit a combination of epithelial and mesenchymal characteristics, referred to as an intermediate or partial EMT state [10,35,36]. This incomplete transition reflects the dynamic and context-dependent nature of EMT in renal pathology [10]. In the present study, chronic PM_2.5_ exposure increased intrarenal expression of TGF-β1, α-SMA, and vimentin. Vitamin D supplementation counteracted the upregulation of α-SMA and TGF-β1. Histological analysis also revealed elevated tubulointerstitial fibrosis scores in PM_2.5_-exposed kidneys. Vitamin D reduced these fibrotic changes. However, the E-cadherin expression was not reduced, suggesting the presence of a partial EMT state. This finding indicates that PM_2.5_ exposure may activate both physiological tissue repair mechanisms and pathological renal fibrogenesis in this experimental model. Consistent with our findings, recent studies have shown that fine PM is associated with the initiation of EMT [5,37]. Maternal PM_2.5_ exposure in mice induced oxidative stress, activated the TGF-β/SMAD3 signaling, and triggered EMT in fetal lung tissue, leading to postnatal pulmonary dysfunction [37]. Similarly, short-term PM_2.5_ exposure has been associated with ongoing pulmonary fibrosis through an oxidative-stress-mediated nuclear factor-κB/inflammation/EMT pathway [38].

RAS activity is a well-established mediator of EMT and plays a critical role in the progression of CKD [9]. Abnormal activation of the RAS can stimulate downstream signaling pathways, such as the TGF-β/Smad3 pathway, which mediates renal fibrosis via EMT [9,39]. Conversely, inhibition of RAS during postnatal renal maturation has been shown to increase renal EMT and induce capillary loss in the glomerular and peritubular regions [40,41]. In addition, aberrant neonatal suppression of intrarenal RAS can result in structural alterations in renal morphology, increasing the susceptibility of adult offspring to hypertension and kidney disease [42,43]. In the context of PM_2.5_ exposure, the biphasic RAS regulation appears particularly significant. Early-life exposure to PM_2.5_ has been shown to suppress RAS activity during critical periods of renal development [16], while prolonged exposure into adulthood may lead to maladaptive overactivation of the RAS cascade. This developmental shift in RAS activity—characterized by neonatal suppression followed by later-life overactivation—likely contributes to the pathogenesis of renal fibrosis and the progression of kidney disease associated with PM_2.5_ exposure. Our study is the first to examine the combined effects of prenatal and postnatal PM_2.5_ exposure on EMT marker expression and TGF-β1 levels in adult rat kidneys. Chronic PM_2.5_ exposure elevated intrarenal α-SMA, vimentin, and TGF-β1 expression, as well as renin activity, supporting a link between PM_2.5_ exposure and fibrogenic signaling. Notably, vitamin D supplementation attenuated these molecular changes, suggesting its protective roles against PM_2.5_ -induced renal damage. However, several limitations should be addressed. First, this study included a relatively small sample size (*n* = 3/group for dams; *n* = 7/group for offspring) and only male rats, excluding potential sex-specific differences. Although this design aimed to minimize the sacrifice of female offspring, it may limit the generalizability of the findings. Nevertheless, accumulating evidence indicates that sex hormones influence the regulation of various physiological systems, and that male offspring may exhibit increased susceptibility to prenatal and perinatal stress under certain conditions [18,19]. These findings imply that sex plays a crucial role in developmental outcomes and highlight the need for future studies to investigate sex-specific responses in similar models. Such efforts may contribute to a deeper understanding of sex-related vulnerabilities in human health, particularly in the context of early-life programming of disease risk. Second, in this study, PM_2.5_ was administered orally, which differs mechanistically from inhalation. Oral exposure may facilitate direct evaluation of PM_2.5_-induced renal toxicity, as ingested particles enter systemic circulation via gastrointestinal absorption and hepatic metabolism, ultimately accumulating in the kidneys. Although inhaled particles are largely eliminated through pulmonary clearance mechanisms, fine particles such as PM_2.5_ can also translocate into the systemic circulation through the alveolar–capillary barrier, reaching distant organs including the kidneys [44,45]. Regardless of the exposure route, the kidneys remain particularly vulnerable to environmental toxins due to their role in concentrating and filtering these substances [46]. Therefore, these findings support the biological plausibility of renal outcomes observed in our oral exposure models. Finally, we have to consider that variance of PM_2.5_ composition may differentially impact the physiological and pathological responses of murine models with specific renal outcomes. Although our current understanding of the toxicology of PM_2.5_ is limited, recent studies have identified potential molecular mechanisms underlying PM_2.5_-induced EMT [5]. Given the inevitable tissue- and cell-type-specific responses to PM_2.5_, the definitive role of PM_2.5_ in renal EMT warrants further investigation and validation.

## 5. Conclusions

In conclusion, both prenatal and postnatal PM_2.5_ exposure exacerbated glomerular and tubular injuries, induced renin expression, and partially activated the EMT response in adult rat kidneys. Vitamin D supplementation alleviated kidney damage associated with chronic PM_2.5_ exposure, providing partial protection against its deleterious effects. While PM_2.5_ exposure and vitamin D administration did not significantly alter intrarenal vitamin D signaling, complex changes in serum calcium, VDR expression, and 25(OH)D levels suggest potential impacts on systemic and intrarenal calcium homeostasis. These findings highlight the need for further research to clarify the mechanisms underlying PM_2.5_-induced renal damage and to develop effective therapeutic strategies.

## Figures and Tables

**Table 1:** Average concentrations and percentages of inorganic ions and WSOC in PM_2.5_ suspensions.

Ion	Liquid Phase Concentration (ppm)	Atmospheric Concentration (μg/m^3^)	%
NO_3_^−^	367	10.4	36.0
WSOC	325	9.4	31.9
NH_4_^+^	137	3.93	13.4
SO_4_^2−^	120	3.47	11.8
Cl^−^	33.3	0.91	3.26
Ca^2+^	15.1	0.24	1.48
Na^+^	12.5	0.31	1.23
K^+^	8.26	0.21	0.81
Mg^2+^	1.05	<0.01	0.1
F^−^	0.49	<0.01	0.05
PO_4_^3−^	0.13	<0.01	0.01
Br^−^	0.09	<0.01	0.009

Atmospheric concentrations of ions and WSOC were measured using an air sampler with a suction volume of 1,380,000 L/day, based on a flow rate of 1000 L/min over a 23 h collection period. The analysis was performed at the Seoul Center of the Korea Basic Science Institute. WSOC, water-soluble organic carbon; PM_2.5_, fine particulate matter.

## Data Availability

The data presented in this study are available on request from the corresponding author.

## Abbreviations

The following abbreviations are used in this manuscript:

ACE	Angiotensin-converting enzyme
AT1R	Angiotensin II type 1-receptor
CKD	Chronic kidney disease
CYP	Cytochrome P450 mixed-function oxidase
ELISA	Enzyme-linked immunosorbent assay
EMT	Epithelial–mesenchymal transition
MT	Masson’s trichrome
NS	Normal saline
PM	Particulate matter
PM_2.5_	Fine particulate matter
PRR	Pro renin receptor
RAS	Renin-angiotensin system
SEM	Standard error of the mean
TGF-β1	Transforming growth factor-β1
VDR	Vitamin D receptor
WSOC	Water-soluble organic carbon
α-SMA	α-smooth muscle actin
